# Surgical considerations and audiological results of cochlear implantation in patients with otosclerosis

**DOI:** 10.3906/sag-1912-238

**Published:** 2020-06-23

**Authors:** Tolgahan ÇATLI, Taşkın TOKAT, Ergül Başaran BOZKURT, Zehra Hilal ADIBELLİ, Uğurtan ERGÜN, Enver ALTAŞ, Levent OLGUN

**Affiliations:** 1 Clinic of Otorhinolaryngology, University of Health Sciences Bozyaka Education and Research Hospital, İzmir Turkey; 2 Clinic of Otorhinolaryngology, Sakarya University Training and Research Hospital, Sakarya Turkey; 3 Clinic of Radiology, University of Health Sciences Bozyaka Education and Research Hospital, İzmir Turkey; 4 Clinic of Otorhinolaryngology, Başkent University Hospital, İzmir Turkey

**Keywords:** Otosclerosis, cochlear implantation, cochlear ossification, facial twitching

## Abstract

**Background/aim:**

To emphasize the role of cochlear implantation (CI) in the auditory rehabilitation of patients with otosclerosis (OS) and share our surgical experiences on this rare group of patients.

**Materials and methods:**

Retrospective analysis of the patients who have a diagnosis of otosclerosis and implanted between January 1998–May 2019 was performed. Preoperative and postoperative clinical, radiological, audiological and surgical findings are presented.

**Results:**

Among 2195 patients who have been implanted in our institution, 12 (0.54%) met the diagnostic criteria of OS according to their preoperative (clinical, radiological, audiological) and peroperative (surgical) findings. Electrode insertion was performed via “round window membrane and cochleostomy” in 8 and 4 patients, respectively. No major complications occured. All patients showed satisfactory performances by means of audiometric scores postoperatively. Nonauditory stimulation (NAS) which manifested as “facial twitching” was a challenging problem in one patient during the surgery and subsided after the operation.

**Conclusion:**

Our experience on CI in patients with OS revealed that the implantation was a relatively safe procedure and had satisfactory impact on audiological performances.

## 1. Introduction

Otosclerosis (OS) is a unique disease of human kind, which primarily results in progressive conductive hearing loss due to ankylosis of stapes footplate to the oval window [1]. Less often the disease may spread to the inner parts of the otic capsule and clinical picture manifest as mixed or sensorineural type profound hearing loss and/or dizziness [2]. Ultrastructural events are mainly centered around the osteoclasts of the otic capsule and their altered cytological activity. Increased bone resorption and formation in the otic capsule end up with mature calcified foci around the footplate, which results in ossicular fixation. Although hearing aids and medical therapy may offer a certain extent of solution in some patients, stapes-oriented surgical interventions are the main treatment options in the audiological rehabilitation of the patients [3,4]. In a group of patients with OS, pure tone air conduction thresholds may exceed 85 dB and these interventions are not able to satisfy patients. In approximately 20%–40% these patients, electrical stimulation of cochlear nerve via cochlear implant has been shown to provide adequate sound perception and communication skills [5]. Despite the fact that cochlear implantation (CI) technology improved over the years, it is still far away being “free of surgical risks” and plenty of complications have been reported in the literature [6]. Ossified cochlea in OS, is an important challenge for the surgeon, which brings along special problems that need to be addressed during or after the implantation procedure [7]. In this retrospective study, we aimed to emphasize the role of CI in the auditory rehabilitation of patients with OS and also shared our surgical experiences on this rare group of patients. 

## 2. Material and methods

The study was carried-out in a tertiary referral center, which had CI experience in 2195 patients including children and adults, over 20 years. Retrospective analysis of the patients who have been diagnosed with OS and implanted between 01 January 1998 and 01 May 2019 was performed. Ethical commitee of the institution approved the study protocol (Protocol number: 03/18). 

### 2.1. Patient selection

Patients who had a history of clinical OS which was supported with audiological and radiological findings and received cochlear implant were included in the study. In our institution decision on CI surgery is made by a committee that composed of at least 3 otolaryngology specialists, 1 audiologist and 1 consultant radiologist when needed. In case of an inadequate amplification via conventional hearing aid (HA), CI was recommended in OS patients. Both thin-slice computed tomography (CT) and Gadolinium-enhanced magnetic resonance imaging (MRI) of the temporal bone were obtained in order to examine the middle ear cleft, cochlear bony structure, cochlear canal patency, inner ear fluids, internal auditory canal contents, cerebellopontine angle and other temporal bone anatomical subsites. CT findings were graded according to the imaging based grading system by Rotteveel et al., where CT records were available (Table 1) [8]. All patients were evaluated by the same test batary, which included “pure tone audiometry (PTA) and tympanometry (TM)” both before and after the procedure. 

**Table 1:** Rotteveel and colleagues’ imaging-based grading systems for otosclerosis.

Type	Otosclerotic lesions of the otic capsule
Type 1	Solely fenestral involvement (thickened footplate and/or narrowed or enlarged windows)
Type 2	Retro-fenestral with or without fenestral involvement
Type 2a	Double ring effect
Type 2b	Narrowed basal turn
Type 2c	Double ring and narrowed basal turn
Type 3	Severe retro-fenestral (unrecognizable otic capsule), with or without fenestral involvement

According to Rotteveel et al [8].

### 2.2. Surgical procedure

All procedures were performed under general anesthesia, by the senior authors of the study at the same institution. Retroauricular approach, simple mastoidectomy and posterior tympanotomy were the basic steps of the surgery. Subperiosteal pocket technique was used to maintain internal receiver stabilization. Electrode insertion routes were scala tympani via round window membrane or cochleostomy. Intraoperative and postoperative complications were also noted. 

### 2.3. Statistical analysis

Statistical analysis of the data was conducted with SPSS 21.0.0 (IBM Corp., Armonk, NY, USA). Pre- and postoperative PTA scores were compared using the Wilcoxon t-test. A “P” value <0.05 was considered statistically significant. 

## 3. Results

Between 01 January 1998 and 01 May 2019, 2195 patients received CI. Among these 2195 patients, 12 (4 females and 8 males) had an etiology of OS, representing 0.54% of all patients implanted during this time. The age at implantation ranged between 44 and 76 years (median, 62 years). The median follow-up time after implantation was 56 months (range, 12–120 months). Patient characteristics (diagnostic criteria such as family history and CT findings) are summarized in Table 2. All patients have used conventional hearing aid (HA) prior to CI surgery (via medical records and telephone questioning). Unfortunately none had a satisfactory results during years especially at the advance stages of their diseases. Concerning about the stapes oriented surgery history of the patients, 5 (S1,S3,S4,S7,S12) had a stapedotomy/stapedectomy procedure before the implantation. However none had a detailed operation note in their medical records but their self reported information suggest that they had some degree of functional benefit from the stapes surgery which is deteriorated during the years mainly attributed to the retrofenestral progression of the disease. Among others with no history of stapes surgery, 4 patients (S2, S5, S9, S10) mentioned that they had informed about the possible stapes surgery candidacy but none had accepted the surgery mainly due to the possibility of loosing their residual hearings after the stapes oriented procedure. In the remaining 3 patients stapes surgery history could not be detailed due to lack of their medical records and/or unsatisfactory patient orientation to their medical past. 

**Table 2:** Diagnostic features.

	S1	S2	S3	S4	S5	S6	S7	S8	S9	S10	S11	S12
Family history	+	+	+	–	+	–	–	–	+	–	–	+
CT grade (Type)(Implanted side/Other side)	1/2a	–	–	–	1/1	1/1	–	2a/2c	2c/2c	1/1	2c/2c	1/1

According to Rotteveel et al [8].

### 3.1. HRCT findings

**Figure 1:**
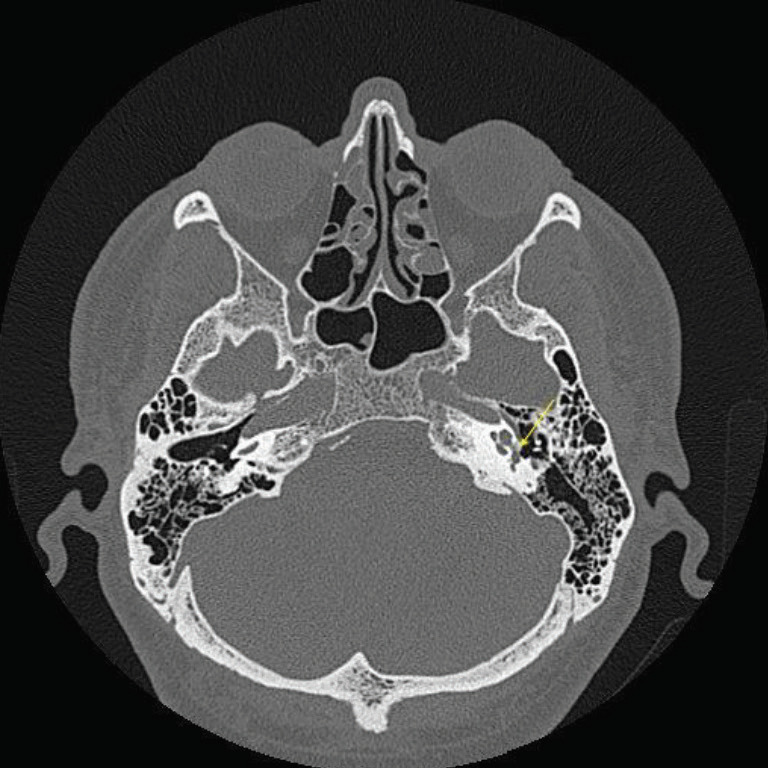
CT scan (axial plane) of patient (S5) shows left sided fenestral otosclerosis (yellow arrow: left sided fenestral involvement).

**Figure 2:**
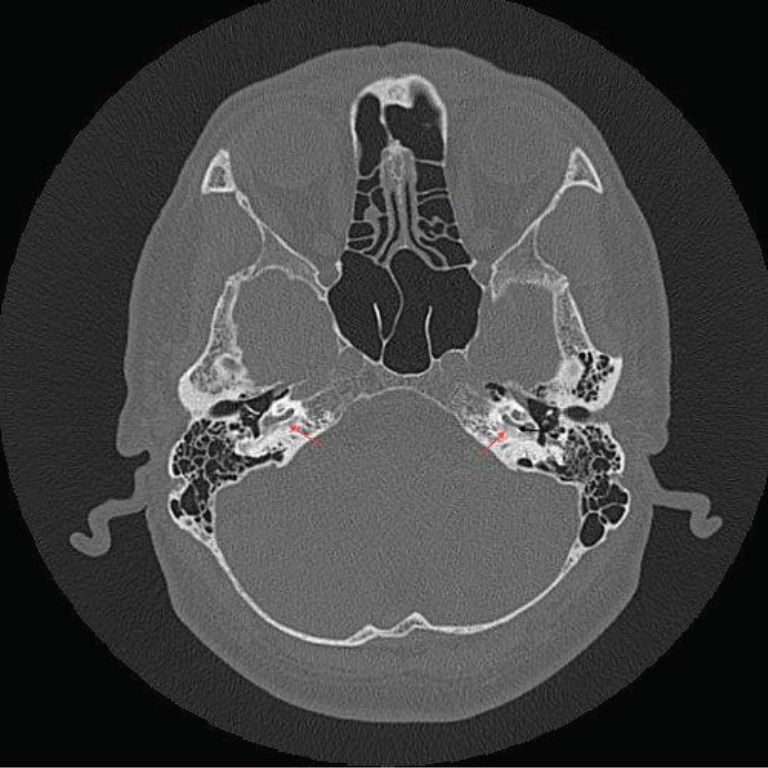
CT scan (axial plane) of patient (S8) shows bilateral retrofenestral otosclerosis (red arrows: bilateral “double ring” ; black arrow: left sided “narrowed cochlear lumen”).

Radiological examinations of temporal bones were performed by a 64-section CT scanner (Aquilion, Canon Medical Systems Corporation, Tochigi, Japan) with 0.6 mm axial section thickness and coronal and sagital reformations at 1 mm. All studies were performed without contrast, and imaging included the entire petrous bone. In 4 of the 12 patients, radiological images were not available to examine and grading through digital screen. However in these 4 patients (S2, S3, S4, S7) radiologist had pointed out some degree of otic capsule density alterations and footplate thickenings in his written reports. According to Rotteveel grading system (16 temporal bones in total), 4 and 2 patients had bilateral “grade 1”(50%) and “grade 2c” (25%) OS, respectively (Figures 1 and 2). The remaining 2 patients had mixed type of OS grades in their ears as summarized in Table 2**. **In patients with bilateral grade 1 OS (S5 ,S6, S10, S12), the main radiological finding were “footplate thickening (S5, S10) and narrowed (S6 )/enlarged (S12) windows”. In patients with bilateral grade 2c (S9, S11), the main radiological finding was “double ring appearance with basal turn narrowing” in both sides. In patients with mixed type of grades, while S1 had footplate thickening in the implanted ear and double ring appearance in the nonimplanted ear, S8 had double ring appearance in the implanted ear and basal turn narrowing in the nonimplanted ear.

### 3.2. Surgical findings

Electrode insertion was performed via “round window (RW) membrane and cochleostomy” in 8 and 4 (S5, S9, S10, and S12) patients respectively. Number of active electrodes for each patient and device characteristics are summarized in Table 3. In 8 of the 12 patients, electrode insertion was performed through the RW. Remaining 4 patients had varying degrees of sclerosis throughout the medial wall of the middle ear cleft (around the RW niche and promontorium) and needed to be drilled to achieve electrode insertion via cochleostomy. In patient S12, anteriorly positioned facial nerve was observed and this was noted as another factor, which has hidden the RW. No major complications occured both intra- and postoperatively. Nonauditory stimulation (NAS), which manifested as “facial twitching” during the surgery, was a challenging problem in one patient (S12) and subsided in the postoperative fitting period. None of the other patients had experienced postoperative facial twitching or other types of NAS. 

### 3.3. Audiological results

A comparison of the PTA scores (dB) before and after the implantation had revealed that the scores were significantly lower after the surgery. While the pure tone thresholds were between 30 dB and 50 dB, discrimination scores were between 70%–90% in the postoperative period [median PTA scores were 100 dB (range, 90–110) and 43 dB (range, 30–50) before and after the implantation, respectively, P = 0.002; median discrimination scores were 16% (range, 12%–20%) and 82% (range, 70%–94%) before and after the implantation, respectively, P = 0.002].

## 4. Discussion

Our experience on CI in patients with cochlear OS revealed that the procedure is relatively safe and effective by means of auditory rehabilitation. Similar to our findings, Ruckenstein et al. showed that these group of patients can express excellent audiological outcomes after the procedure. In their study (n = 8) all patients had expressed significantly higher scores on Central Institute for the Deaf (CID) sentence test after CI [9]. In our study group, we had also applied speech tests (bi-syllable open set test; language specific sentence test) to some of our recipients both before and after the procedure. However, results of these tests are not sufficient to draw a precise statistical conclusion and this might be considered as a limitation of our study. 

While unaffected neural element is the key factor for adequate electrical stimulation of the auditory pathways, patients with cochlear OS seem to have relatively favorable cochlear status compared to patients who have deseased cochleas. Therefore, excellent auditory perception skills seem to be related to the unique pathology of OS which typically spares medial aspect of the cochlea while damaging its lateral wall [9,10].

Since the diagnostic sensitivity of CT scanning of the temporal bone in OS is not so high it is not possible to exclude OS when demineralization is not present in CT [8,11]. In our study group, 8 patients showed varying degrees of otic capsule demineralization and other features of retrofenestral involvement. Among these 8 patients, 6 (75%) and 2 patients (25%) had symmetrical (same grade) and asymmetrical involvement respectively. In their study group Rotteveel et al. reported 20% symmetrical involvement [8]. In another study, Ruckenstein et al. reported 50% ottic capsule involvement as “rarefaction of otic capsule bone, osteoneogenesis within the cochlear ducts” in their patient group. However the bilaterality and/or symmetricity of the involvement were not clarified in the article [9]. In our remaining 4 patients, CT images were not available but radiological reports have pointed out varying degrees of demineralization and hypodensity of the otic capsule and also fenestral involvement. In these 4 patients, electrode insertion was achieved via round window membrane and fenestral exposure was satisfactory. We suggest this could be related to “false positive” radiological evaluation or very early stage of the disease process in these 4 patients. However, we cannot make a certain conclusion due to the unavailability of the images. This might be considered as another limitation of our study. Concerning about the “false positive” results in the radiological evaluation of OS, we might consider 3 of our patients (S1, S6, and S11) had false positive results in CT imaging. Although radiologist reported “fenestral OS” (S1 and S6: Rotteveel grade 1; S11: Rotteveel grade 2c) in these 3 patients, round window exposure through the posterior tympanotomy was achieved and electrode insertion was performed via RW membrane to the inner ear.

In 4 patients (S5, S9, S10, S12), RW niche could not be identified due to the sclerotic lesions located around the niche. Therefore, electrode insertion was performed via cochleostomy. Previously, Ruckenstein et al. and Fayad et al. reported in their studies that they needed some degree of cochlear basal turn drillings in order to eradicate sclerotic lesions and achieve patent cochlear lumen [9,12]. However, we did not need drill-out procedure even in patients with narrowed cochlear lumen (S9,S11). The classical appearance of retrofenestral OS on CT is a pericochlear hypodensity named “double-ring” (aka 4th ring of Valvassori) or “halo sign”, which is highly characteristic for cochlear OS [13]. In our study group, halo sign was present bilaterally in 3 patients (S8, S9, S11) and unilaterally in 1 patient (unimplanted ear). Two (S9, S11) out of these 3 patients had required cochleostomy. According to our experience, it is possible to conclude that if a patient has halo sign in CT imaging, a cochleostomy is more likely to be needed. This might be considered as another clinical significance of halo sign in the CI of patients with OS. There is no doubt that larger studies are necessary to make stronger and statistically significant conclusions regarding this issue.

Facial nerve stimulation (FNS) after CI is a rare but potentially devastating problem [14]. The reported incidence of this phenomenon in the literature varies between 0.9% to 14.6% [15,16]. FNS is more frequent when the recipient has cochlear OS. This is mainly due to the altered otic capsule bony architect after a process of demineralization and sclerosis. Thus, electric current becomes more dispersible as a result of decreased electrical impedance of the bone and the reduced distance between the electrode and the facial nerve due to bone loss and cavity formation [17]. There are some options to alleviate postoperative FNS such as “using triphasic pulse patterns (TPP)”, “deactivation of the offending electrodes” or “prolonging the phase duration while reducing the amplitude to keep the total charge constant but limiting the current spread” [18]. In our one patient experience, we used TPP as a relatively novel option and obtained a satisfactory result. 

In conclusion, our experience on CI in patients with OS revealed that the implantation is a relatively safe procedure and had a satisfactory impact on audiological performances. Diversity of the cochlear anatomy due to ossification process should be kept in mind and surgeons need to be prepared for an alternative insertion scenario during the procedure.

## Acknowledgment

No funding has been received for this study.

## Conflict of interest

The authors declare no conflict of interest.
